# Glycated albumin levels are associated with adverse stroke outcomes in patients with acute ischemic stroke in China

**DOI:** 10.1111/1753-0407.13600

**Published:** 2024-09-12

**Authors:** Jiawen Mao, Meng Wang, Chunjuan Wang, Hongqiu Gu, Xia Meng, Yong Jiang, Xin Yang, Jing Zhang, Yunyun Xiong, Xingquan Zhao, Liping Liu, Yilong Wang, Yongjun Wang, Zixiao Li, Bihong Zhu

**Affiliations:** ^1^ Department of Neurology, Beijing Tiantan Hospital Capital Medical University Beijing China; ^2^ China National Clinical Research Center for Neurological Diseases Beijing Tiantan Hospital Beijing China; ^3^ National Center for Healthcare Quality Management in Neurological Diseases Beijing Tiantan Hospital Beijing China; ^4^ Research Unit of Artificial Intelligence in Cerebrovascular Disease Chinese Academy of Medical Sciences Beijing China; ^5^ Department of Neurology Huangyan Hospital of Wenzhou Medical University Zhejiang China

**Keywords:** diabetes, glycated albumin, poststroke outcome, stroke

## Abstract

**Background and Aim:**

Glycated albumin (GA) is a biomarker monitoring glycemia 2–4 weeks before stroke onset. This study was designed to explore the association between GA levels with poststroke outcomes in patients with acute ischemic stroke or transient ischemic attack (TIA).

**Method:**

Participants with ischemic stroke or TIA who had a baseline GA measurement were included in the Third China National Stroke Registry study. The effect of GA on stroke recurrence, poor functional outcomes, and combined vascular events was examined during the 1‐year follow‐up period. Multivariate Cox and logistic regression models were performed to evaluate the association. Discrimination tests were used to examine the incremental predictive value of GA when incorporating it into the conventional model.

**Results:**

A total of 3861 participants were enrolled. At the 3‐month follow‐up, the elevated GA level was associated with an increased risk of poor functional outcomes (adjusted odds ratio [OR], 1.45; 95% confidence interval [CI], 1.01–2.09). A similar increase was observed for stroke recurrence (adjusted hazard ratio [HR], 1.56; 95% CI, 1.09–2.24), poor functional outcomes (adjusted OR, 1.62; 95% CI, 1.07–2.45), and combined vascular events (adjusted HR, 1.55; 95% CI, 1.09–2.20) at the 1‐year follow‐up. In nondiabetic patients, the association between GA and poor functional outcomes was more pronounced (adjusted OR, 1.62; 95% CI, 1.05–2.50). Adding GA into the conventional model resulted in slight improvements in predicting poor functional outcomes (net reclassification improvement [NRI]: 12.30% at 1 year).

**Conclusion:**

This study demonstrated that elevated GA levels in serum were associated with stroke adverse outcomes, including stroke recurrence, poor functional outcomes, and combined vascular events, in patients with ischemic stroke or TIA.

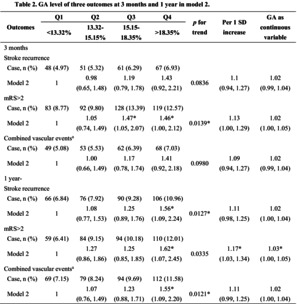

## Graphical abstract

Highlights

- Elevated GA levels in serum were associated with stroke adverse outcomes.
- Adding GA into the conventional model resulted in slight improvements in predicting poor functional outcomes.
- The association between GA and poor functional outcomes was more pronounced in nondiabetic patients.

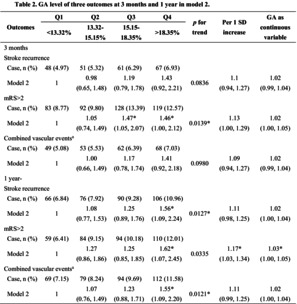

## 1 INTRODUCTION

Stroke is a major cerebrovascular disease with high incidence, disability rate, and mortality rate.^1^ Previous studies have shown that 15%–30% of patients with ischemic stroke have poor functional outcomes.^2^ Approximately 20% of patients may experience recurrent stroke, which can deteriorate functional outcomes and even increase mortality.^3^, ^4^, ^5^ Therefore, accurate risk prediction of recurrence and unfavorable functional outcomes is necessary to optimize stroke care. Blood markers are objective predictive factors that can increase the predictive accuracy of scores.^6^

Diabetes mellitus characterized by hyperglycemia is a risk factor for stroke and can lead to the occurrence of adverse and poor functional outcomes.^7^ Data from the Chinese Stroke Center Alliance between 2015 and 2019 showed that patients with diabetes had a 30% increased risk of all‐cause death and an 8% increased risk of major adverse cardiovascular events.^8^ Glycated hemoglobin (HbA1c) is considered the gold standard for glycemic control and reflects the glycemia level over the past 2–3 months. However, it does not reflect a shorter period glycemic situation before stroke onset, and its predictive effect on adverse stroke outcomes remains controversial.^9^ Glycated albumin (GA) is a relatively novel blood indicator that reflects blood glucose levels 2–4 weeks before stroke onset, and it is closely associated with the development of diabetes. One study reported that higher GA levels in a healthy population were associated with the incidence of stroke and cardiovascular events.^10^ However, limited studies have focused on GA and adverse stroke outcomes.

The aim of this study was to validate the predictive role of GA in poststroke prognosis, including stroke recurrence, poor functional outcomes, and combined vascular events at 3 months and 1 year in the Third China National Stroke Registry (CNSR‐III).

## 2 PATIENTS AND METHODS

### 2.1 Population and patients

The current study derived data from the CNSR‐III, a registry study recruiting patients with acute ischemic cerebrovascular events between August 2015 and March 2018 in China. Patients were enrolled consecutively from 201 hospitals with the following criteria: (1) age ≥18 years; (2) diagnosed with ischemic stroke or transient ischemic attack (TIA) confirmed by magnetic resonance imaging (MRI) or brain computed tomography (CT); (3) within 7 days from the onset of symptoms to enrollment; and (4) voluntary informed consent. Patients were excluded if they met any of the following criteria: (1) lacked serum GA data at baseline; (2) were lost to follow‐up and admission time; and (3) were missing admission time. The present study was performed in accordance with the guidelines described by the Helsinki Declaration and was approved by the ethics committees of Beijing Tiantan Hospital (No. KY2015‐001‐01).

### 2.2 Patient characteristics and outcome measurements

Baseline information was collected by well‐trained neurologists at each participating center by face‐to‐face interviews or medical records, including baseline data (age, sex, drinking, and smoking status), medical histories (stroke, hypertension, cardiovascular disease, coronary heart disease, atrial fibrillation, dyslipidemia, diabetes mellitus), the modified Rankin Scale (mRS) score before stroke, and the National Institutes of Health Stroke Scale (NIHSS) score. Moreover, intravenous thrombolysis therapy and the TOAST (Trial of Org 10172 in Acute Stroke Treatment) criteria were also assessed. Fasting blood samples were collected within 24 h of admission from 171 study sites and delivered through a cold chain to Beijing Tiantan Hospital. All blood samples were collected in Ethylene Diamine Tetraacetic Acid anticoagulant collection tubes and stored at −80°C in a refrigerator. The laboratory personnel were blinded to the serum specimens, clinical information, and outcomes.

Stroke recurrence, poor functional outcomes, and combined vascular events were the primary outcomes. Stroke recurrence was defined as a new focal neurological impairment that was confirmed by neuroimaging, including both ischemic stroke and hemorrhagic stroke. An mRS score >2 was defined as poor functional outcome. The combined vascular events included cardiovascular death, nonfatal myocardial infarction, and nonfatal stroke. The outcomes mentioned above were observed by face‐to‐face interview at 3 months and by telephone at 1 year after stroke onset.

### 2.3 Statistical analysis

Patients participating in this study were assigned to four groups according to the quartiles of GA assessed on admission. Continuous variables conforming to a normal distribution are presented as the means ± standard deviations (SDs) and were compared using Analysis of Variance. Skewed continuous variables that did not exhibit a normal distribution are presented as medians with interquartile ranges (IQRs) and were compared using the Kruskal–Wallis *U* test. Categorical variables are shown as numbers (proportions) and were compared using the χ^2^‐test or Fisher's exact test.

The association of GA levels with stroke recurrence and combined vascular events was estimated using a multivariate Cox regression model, and poor functional outcomes were analyzed by a logistic regression model. Hazard ratios (HRs) or odds ratios (ORs) with 95% confidence intervals (CIs) were calculated after adjusting for potential confounding factors. Three steps were performed to adjust the covariates: The first model was a crude model without adjustment. In the second model, age and sex were added. In the third model, smoking and drinking status, medical histories (including stroke, hypertension, cardiovascular disease, coronary heart disease, atrial fibrillation, dyslipidemia, and diabetes mellitus), the NIHSS score, the mRS score before stroke, the TOAST classification (including five subtypes of ischemic stroke: large‐artery atherosclerosis, cardioembolism, small‐vessel occlusion, other determined etiologies, and undetermined causes), intravenous thrombolysis therapy, and laboratory tests (including hypersensitive C‐reactive protein [hsCRP] and low‐density lipoprotein [LDL]) were added.

In addition, discrimination tests (C‐statistics, net reclassification improvement [NRI] and integrated discrimination improvement [IDI]) were used to evaluate whether GA improved risk prediction for stroke adverse outcomes in the conventional prediction model, including age, sex, smoking and drinking status, medical histories, NIHSS score, mRS score before stroke, TOAST classification, intravenous thrombolysis therapy and serum levels of hsCRP, and LDL. Restricted cubic spline analysis was used to address the association. Then, the interaction of age (<65 or ≥65 years), history of diabetes, and sex was examined by subgroup analysis. All statistical analyses were conducted using SAS version 9.4 (SAS Institute Inc.).

## 3 RESULTS

### 3.1 Baseline characteristics

**FIGURE 1:**
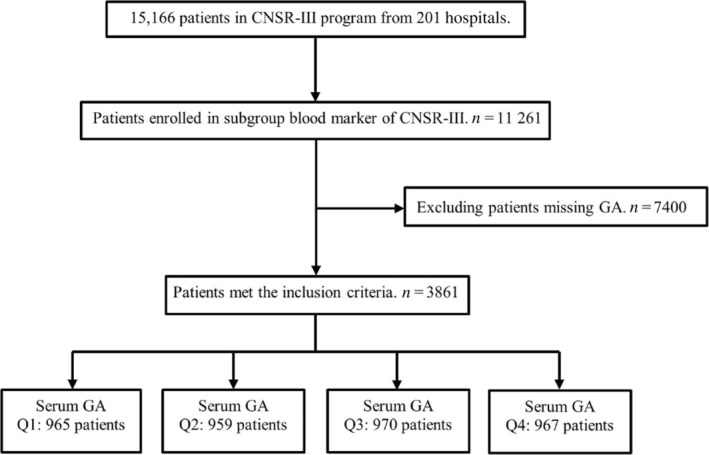
Flow diagram of patient selection. CNSR‐III, Third China National Stroke Registry; GA, glycated albumin; Q, quartile.

Figure 1 shows the process of enrollment: GA was measured as a part of the protocol in blood marker subgroup, where 3861 participants had baseline GA measurements and completed the 1‐year follow‐up, and 7400 were excluded due to missing data. The baseline characteristics of the study population according to serum GA levels are shown in Table 1. The participants were categorized into four groups according to quartiles of GA at baseline (<13.32%; 13.32%–15.15%; 15.15%–18.35%; >18.35%). We found that patients with higher GA were predominantly male and elderly overall and more likely to have a history of diabetes, stroke, hypertension, dyslipidemia, and cardiovascular disease.

**TABLE 1:** Demographic and clinical characteristics.

Characteristic	Total	Q1	Q2	Q3	Q4	*p* value
		<13.32%	13.32%–15.15%	15.15%–18.35%	>18.35%	
*N* (%)	3861	965 (24.99)	959 (24.84)	970 (25.12)	967 (25.05)	
Anthropometric information						
Age, (years)	62.73 ± 11.17	58.71 ± 11.52	62.6 ± 10.79^a^	65.24 ± 10.79^a,b^	64.36 ± 10.44^a,b^	<0.0001
Male, *n* (%)	2595 (67.21)	702 (72.75)	652 (67.99)	608 (62.68)^a^	633 (65.46)^a^	<0.0001
Smoking, *n* (%)	1173 (30.38)	390 (40.41)	302 (31.49)^a^	237 (24.43)^a,b^	244 (25.23)^a,b^	<0.0001
Drinking, *n* (%)	514 (13.31)	185 (19.17)	114 (11.89)^a^	111 (11.44)^a^	104 (10.75)^a^	<0.0001
Time between the onset of the symptoms and the enrollment, (days)	1.10 ± 1.36	1.06 ± 1.33	1.10 ± 1.37	1.06 ± 1.33	1.20 ± 1.43	0.1146
Prestroke physical activity, *n* (%)	2284 (59.16)	570 (24.96)	542 (23.73)	586 (25.66)	586 (25.66)	0.2372
Medical history						
Diabetes mellitus, *n* (%)	948 (24.55)	52 (5.39)	77 (8.03)	211 (21.75)^a,b^	608 (62.87)^a,b,c^	<0.0001
Stroke, *n* (%)	907 (23.49)	173 (17.93)	248 (25.86)^a^	244 (25.15)^a^	242 (25.03)^a^	0.0001
Hypertension, *n* (%)	2414 (62.52)	561 (58.13)	584 (60.9)	633 (65.26)^a^	636 (65.77)^a^	0.0009
Dyslipidemia, *n* (%)	274 (7.10)	62 (6.42)	47 (4.90)	74 (7.63)	91 (9.41)^b^	0.0012
Cardiovascular disease, *n* (%)	529 (13.70)	88 (9.12)	118 (12.30)	163 (16.80)^a,b^	160 (16.55)^a,b^	<0.0001
Coronary heart disease, *n* (%)	402 (10.41)	63 (6.53)	87 (9.07)	118 (12.16)^a^	134 (13.86)^a,b^	<0.0001
Atrial fibrillation, *n* (%)	258 (6.68)	53 (5.49)	62 (6.47)	84 (8.66)^a^	59 (6.10)	0.0311
Prestroke medication history						
Antiplatelet therapy, *n* (%)	684 (17.72)	113 (2.93)	188 (4.87)^a^	190 (4.92)^a^	193 (5.00)^a^	<0.0001
Anticoagulation treatment, *n* (%)	25 (0.65)	6 (0.16)	9 (0.23)	7 (0.18)	3 (0.08)	0.3817
Hypoglycemic treatment, *n* (%)	734 (19.01)	36 (0.93)	52 (1.35)	158 (4.09) ^a,b^	488 (12.64)^a,b,c^	<0.0001
Insulin	264 (35.97)	10 (1.36)	7 (0.95)	38 (5.18)	209 (28.47)	<0.0001
Biguanides	337 (45.91)	18 (2.45)	28 (3.81)	86 (11.72)	205 (27.93)	0.0271
Sulfonylureas	139 (18.94)	7 (0.95)	15 (2.04)	33 (4.50)	84 (11.44)	0.1978
Thiazolidinediones	9 (1.23)	0 (0.00)	0 (0.00)	2 (0.27)	7 (0.95)	0.7364

Clinical evaluation						
Intravenous thrombolysis, *n* (%)	341 (8.83)	99 (10.26)	93 (9.70)	80 (8.25)	69 (7.14)	0.0657
TOAST type, *n* (%)						
Large‐artery atherosclerosis	1010 (26.16)	237 (24.56)	252 (26.28)	231 (23.81)^a^	290 (29.99)^a^	0.0008
Cardiogenic embolism	234 (6.06)	49 (5.08)	55 (5.74)	71 (7.32)^a^	59 (6.10)^a^	0.0008
Small artery occlusion	823 (21.32)	180 (18.65)	194 (20.23)	236 (24.33)^a^	213 (22.03)^a^	0.0008
Other determined etiology	49 (1.27)	16 (1.66)	14 (1.46)	11 (1.13)^a^	8 (0.83)^a^	0.0008
Undetermined cause	1745 (45.20)	483 (50.05)	444 (46.30)	421 (43.40)^a^	397 (41.05)^a^	0.0008
NIHSS at admission, median (IQR)	3 (1–6)	3 (1–6)	3 (1–6)	3 (1–6)	3 (2–6)	0.1661
Prestroke mRS score ≤1, *n* (%)	3518 (91.12)	888 (92.02)	859 (89.57)	887 (91.44)	884 (91.42)	0.3863
Laboratory tests						
Glycated albumin, %	15.15 ± 5.03	12.24 ± 1.45	14.19 ± 0.91^a^	16.41 ± 1.61^a,b^	22.78 ± 7.67^a,b,c^	<0.0001
Fasting blood glucose, mmol/L	3081 (83.70)	5.37 ± 1.19	5.43 ± 1.30^a^	5.93 ± 1.71^a,b^	9.03 ± 3.46^a,b,c^	<0.0001
Glycated hemoglobin, %	2429 (62.91)	5.63 ± 0.87	5.78 ± 0.84	6.14 ± 1.00^a,b^	8.38 ± 2.11^a,b,c^	<0.0001
Hemoglobin, g/L	3818 (98.89)	144.37 ± 16.84	139.20 ± 16.12^a^	137.14 ± 15.81^a^	139.59 ± 16.26^a,c^	<0.0001
Blood platelet, ×10^9^/L	3813 (98.76)	227.87 ± 65.71	218.39 ± 69.80^a^	211.61 ± 59.90^a^	208.09 ± 62.01^a,b^	<0.0001
White blood cells, ×10^9^/L	3817 (98.86)	7.47 ± 2.44	7.05 ± 2.07	10.22 ± 99.54	7.25 ± 2.19	0.4634
hsCRP, mg/L	3860 (99.98)	7.09 ± 29.07	6.32 ± 21.47	7.58 ± 27.38	6.71 ± 19.08	0.7074
LDL, mmol/L	3766 (97.54)	2.41 ± 0.90	2.27 ± 0.82^a^	2.45 ± 0.99^b^	2.46 ± 1.01^b^	<0.0001

*Note*: Variables are expressed as median(s) or percentages. Glycated albumin (GA) was expressed as quartiles (Q1, <13.32%; Q2, 13.32%–15.15%; Q3, 15.15%–18.35%; Q4, ≥18.35%). *a*, *b* and *c* stand for statistically significant in multiple comparisons, *p* < 0.0083. *a* stands for comparison to group Q1; *b* stands for comparison to group Q2; and *c* stands for comparison to group Q3.

Abbreviations: hsCRP, hypersensitive C‐reactive protein; IQR, interquartile range; LDL, low‐density lipoprotein; Q, quartile; mRS, modified Rankin Scale; NIHSS, the National Institutes of Health Stroke Scale; TOAST, Trial of Org 10 172 in Acute Stroke Treatment.

### 3.2 GA and clinical outcomes

At the 3‐month follow‐up, 227 (6.17%) patients had stroke recurrence, 442 (10.93%) had poor functional outcomes, and 232 (6.01%) had combined vascular events. During the 1‐year follow‐up, 347 (8.99%), 338 (8.75%), and 354 (9.17%) patients experienced poor functional outcomes, stroke recurrence, and combined vascular events, respectively.

**FIGURE 2:**
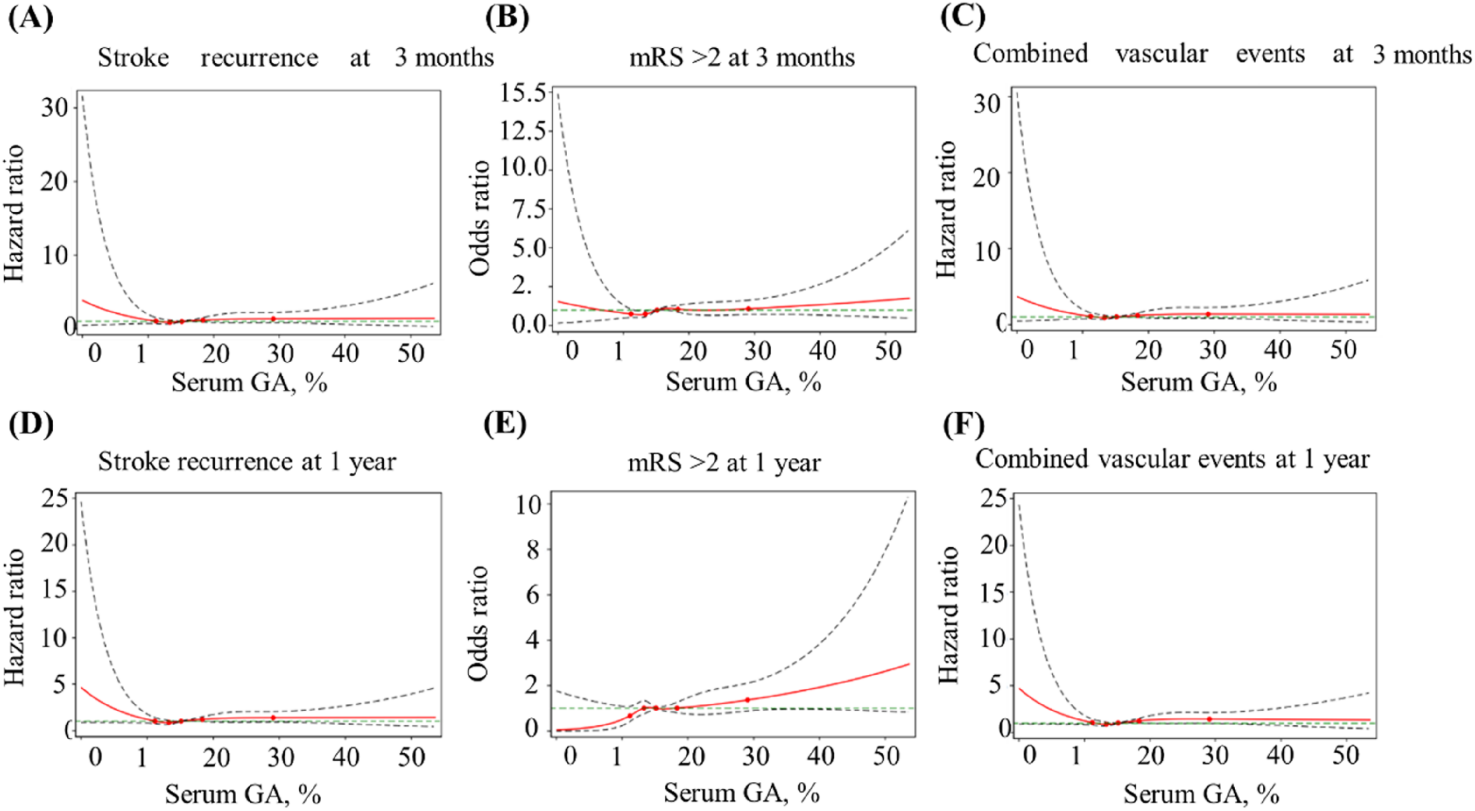
Spline models of the association between glycated albumin (GA) and stroke adverse outcomes. The association between GA levels and (A) stroke recurrence, (B) modified Rankin Scale (mRS) score >2, and (C) combined vascular events at 3 months is shown in the first line, and the relationship between GA and (D) stroke recurrence, (E) mRS score >2, and (F) combined vascular events at the 1‐year follow‐up is shown in the second line. The hazard ratios from the Cox regression model were adjusted for the variables in model 2 in Table 2. The red lines indicate the adjusted hazard ratio, and the green lines indicate the 95% confidence interval.

Regarding poor functional outcomes, a continuous GA value was significantly associated with functional deterioration both at 3 months (crude OR, 1.02; 95% CI, 1.00–1.04) and 1 year (crude OR, 1.03; 95% CI, 1.01–1.05) after stroke onset in the unadjusted model. When GA was classified into quartiles, patients with the GA level in quartile 4 had a higher risk of adverse functional outcomes than those with in GA in quartile 1, which showed a 49% (crude OR, 1.49; 95% CI, 1.11–2.01) increase at 3 months and a 99% (crude OR, 1.99; 95% CI 1.43–2.77) increase at 1 year. Moreover, the association persisted after adjusting for potential confounders at both 3 months (adjusted OR, 1.46; 95% CI, 1.00–2.12) and 1 year (adjusted OR, 1.62; 95% CI, 1.07–2.45). Similar results were observed for stroke recurrence and combined vascular events. At the 1‐year follow‐up, the incidence of stroke recurrence and combined vascular events was increased in patients with higher GA levels, which revealed crude HRs of 1.63 (95% CI, 1.20–2.21) and 1.65 (95% CI, 1.22–2.22), respectively. The adjusted models also showed the same increasing trend that was statistically significant. In the 3‐month model, the higher GA group had a higher trend of stroke recurrence and combined vascular events, but there was no significant difference in HRs (Table 2). Multivariable‐adjusted restricted cubic spline analyses showed that with the increase in GA level, the occurrence of adverse outcomes, including stroke recurrence, poor functional outcomes, and combined vascular events, showed an increasing trend. However, there was no statistical significance (Figure 2).

**TABLE 2:** GA level of three outcomes at 3 months and 1 year for ischemic stroke patients.

Outcomes	Q1	Q2	Q3	Q4	*p* for trend	Per 1 SD increase	GA as continuous variable
	<13.32%	13.32%–15.15%	15.15%–18.35%	>18.35%			
3 months							
Stroke recurrence							
Case, *n* (%)	48 (4.97)	51 (5.32)	61 (6.29)	67 (6.93)			
Unadjusted	1	1.07 (0.72–1.59)	1.28 (0.87–1.86)	1.40 (0.97–2.03)	0.0456*	1.08 (0.96–1.22)	1.01 (0.99–1.04)
Model 1	1	1.03 (0.69–1.53)	1.19 (0.81–1.75)	1.32 (0.91–1.93)	0.1013	1.07 (0.94–1.21)	1.01 (0.99–1.03)
Model 2	1	0.98 (0.65–1.48)	1.19 (0.79–1.78)	1.43 (0.92–2.21)	0.0836	1.1 (0.94–1.27)	1.02 (0.99–1.04)
Model 3	1	0.95 (0.61–1.46)	1.18 (0.78–1.80)	1.34 (0.82–2.19)	0.1815	1.05 (0.88–1.25)	1.01 (0.98–1.04)
mRS score >2							
Case, *n* (%)	83 (8.77)	92 (9.80)	128 (13.39)	119 (12.57)			
Unadjusted	1	1.13 (0.83–1.54)	1.61* (1.2–2.15)	1.49* (1.11–2.01)	0.0011*	1.11* (1.01–1.22)	1.02* (1.00–1.04)
Model 1	1	1.00 (0.73–1.38)	1.32 (0.98–1.78)	1.27 (0.94–1.72)	0.0412*	1.08 (0.98–1.19)	1.01 (1.00–1.03)
Model 2	1	1.05 (0.74–1.49)	1.47* (1.05–2.07)	1.46* (1.00–2.12)	0.0139*	1.13 (1.00–1.29)	1.02 (1.00–1.05)
Model 3	1	0.95 (0.66–1.37)	1.35 (0.95–1.93)	1.36 (0.9–2.06)	0.0463*	1.13 (0.98–1.30)	1.02 (1.00–1.05)
Combined vascular events^a^							
Case, *n* (%)	49 (5.08)	53 (5.53)	62 (6.39)	68 (7.03)			
Unadjusted	1	1.09 (0.74–1.61)	1.27 (0.87–1.85)	1.39 (0.97–2.01)	0.0512	1.08 (0.95–1.21)	1.01 (0.99–1.03)
Model 1	1	1.04 (0.70–1.53)	1.17 (0.80–1.71)	1.3 (0.89–1.88)	0.1299	1.06 (0.94–1.20)	1.01 (0.99–1.03)
Model 2	1	1.00 (0.66–1.49)	1.17 (0.78–1.74)	1.41 (0.92–2.18)	0.0980	1.09 (0.94–1.27)	1.02 (0.99–1.04)
Model 3	1	0.96 (0.63–1.48)	1.16 (0.77–1.76)	1.31 (0.81–2.14)	0.2164	1.05 (0.88–1.24)	1.01 (0.98–1.04)
1 year							
Stroke recurrence							
Case, *n* (%)	66 (6.84)	76 (7.92)	90 (9.28)	106 (10.96)			
Unadjusted	1	1.16 (0.83–1.61)	1.37 (1.00–1.88)	1.63* (1.2–2.21)	0.0009*	1.12* (1.02–1.23)	1.02* (1.00–1.04)
Model 1	1	1.1 (0.79–1.54)	1.26 (0.91–1.74)	1.51* (1.11–2.07)	0.0052	1.1* (1.00–1.22)	1.02* (1.00–1.03)
Model 2	1	1.08 (0.77–1.53)	1.25 (0.89–1.76)	1.56* (1.09–2.24)	0.0127*	1.11 (0.98–1.25)	1.02 (1.00–1.04)
Model 3	1	0.98 (0.69–1.41)	1.25 (0.88–1.77)	1.50* (1.03–2.18)	0.0201*	1.08 (0.95–1.23)	1.01 (0.99–1.04)
mRS score >2							
Case, *n* (%)	59 (6.41)	84 (9.15)	94 (10.18)	110 (12.01)			
Unadjusted	1	1.47* (1.04–2.08)	1.66* (1.18–2.33)	1.99* (1.43–2.77)	<0.0001*	1.18* (1.07–1.3)	1.03* (1.01–1.05)
Model 1	1	1.23 (0.86–1.74)	1.21 (0.85–1.72)	1.55* (1.11–2.18)	0.0135*	1.15* (1.04–1.28)	1.02* (1.01–1.04)
Model 2	1	1.27 (0.86–1.86)	1.25 (0.85–1.85)	1.62* (1.07–2.45)	0.0335	1.17* (1.03–1.34)	1.03* (1.00–1.05)
Model 3	1	1.23 (0.82–1.84)	1.25 (0.83–1.87)	1.69* (1.10–2.60)	0.0239*	1.22* (1.06–1.40)	1.04* (1.01–1.06)
Combined vascular events^a^							
Case, *n* (%)	69 (7.15)	79 (8.24)	94 (9.69)	112 (11.58)			
Unadjusted	1	1.15 (0.83–1.59)	1.37* (1.00–1.87)	1.65* (1.22–2.22)	0.0005*	1.12* (1.03–1.23)	1.02* (1.00–1.04)
Model 1	1	1.08 (0.78–1.50)	1.24 (0.90–1.70)	1.51* (1.11–2.05)	0.0043*	1.11* (1.01–1.22)	1.02* (1.00–1.04)
Model 2	1	1.07 (0.76–1.49)	1.23 (0.88–1.71)	1.55* (1.09–2.20)	0.0121*	1.11 (0.99–1.25)	1.02 (1.00–1.04)
Model 3	1	0.98 (0.69–1.39)	1.20 (0.85–1.69)	1.47* (1.02–2.13)	0.0254*	1.09 (0.96–1.23)	1.01 (0.99–1.04)

*Note*: Hazard ratios (HRs) with 95% confidence intervals (CIs) were used for stroke recurrence and combined vascular events; Odds ratios (ORs) with 95% CIs were used for modified Rankin Scale (mRS) score >2. Unadjusted model was crude model without adjustment. Model 1 was adjusted for age and sex. Model 2 was adjusted for age, sex, smoking and drinking status, medical histories (including stroke, hypertension, cardiovascular disease, coronary heart disease, atrial fibrillation, dyslipidemia, diabetes mellitus), the National Institutes of Health Stroke Scale (NIHSS) score, the mRS score before stroke, the TOAST (Trial of Org 10172 in Acute Stroke Treatment) classification (including five subtypes of ischemic stroke: large‐artery atherosclerosis, cardioembolism, small‐vessel occlusion, other determined etiologies, and undetermined causes), intravenous thrombolysis therapy and laboratory tests (including hypersensitive C‐reactive protein and low‐density lipoprotein). Model 3 was adjusted for age, sex, smoking and drinking status, medical histories (including stroke, hypertension, cardiovascular disease, coronary heart disease, atrial fibrillation, dyslipidemia, diabetes mellitus), the NIHSS score, the mRS score before stroke, the TOAST classification (including five subtypes of ischemic stroke: large‐artery atherosclerosis, cardioembolism, small‐vessel occlusion, other determined etiologies, and undetermined causes), intravenous thrombolysis therapy and laboratory tests (including hypersensitive C‐reactive protein and low‐density lipoprotein), the time between the onset of the symptoms and the enrollment, prestroke physical activity, and prestroke medication history (including antiplatelet therapy, anticoagulation therapy, and hypoglycemic treatment).

Abbreviations: GA, glycated albumin; SD, standard deviation.

^a^ Combined vascular events included cardiovascular death, nonfatal myocardial infarction, and nonfatal stroke.

* *p* < 0.05.

In subgroup analyses, GA was associated with stroke recurrence (adjusted HR, 1.69; 95% CI, 1.12–2.54), poor functional outcomes (adjusted OR, 1.82; 95% CI, 1.12–2.94), and combined vascular events (adjusted HR, 1.82; 95% CI, 1.12–2.94) only in nondiabetic patients at 1 year after adjusting for confounders. There was no statistical significance between GA and stroke outcomes in patients with diabetes. For the age subgroup, there was a stronger association between GA and stroke outcomes in patients younger than 65 years, with an adjusted HR of 2.03 (95% CI, 1.25–3.32) for stroke recurrence, an OR of 2.28 (95% CI, 1.20–4.32) for poor functional outcomes, and an HR of 1.96 (95% CI, 1.21–3.17) for combined vascular events. There was no statistical significance between GA and stroke outcomes in the sex subgroup (Table S1).

### 3.3 Incremental predictive value of GA

In addition, we conducted the discrimination test and found a slight improvement in predicting the risk of poor functional outcomes at 3 months and 1 year after stroke onset. After adding GA into model 2, the NRI of poor functional outcomes was 18.67% (*p* = 0.0004) at 3 months and 12.30% (*p* = 0.03) at 1 year (Table 3). However, there was no statistical significance in the C‐statistic and IDI analysis. The accuracy, specificity, sensitivity, and predictive values of C‐statistic are shown in Table s2.

**TABLE 3:** Reclassification and disclination statistics for stroke adverse outcomes when adding to GA levels.

Clinical outcomes	Model	C‐statistic		NRI		IDI	
		Estimate (95% CI)	*p* value	Estimate (95% CI)	*p* value	Estimate (95% CI)	*p* value
3 months							
Stroke recurrence	Conventional model^a^	0.591 (0.550 to 0.632)	0.7724	Ref.	0.193	Ref.	0.1200
	Conventional model +GA	0.593 (0.551 to 0.634)		0.0480 (−0.0390 to 0.1180)		0.000 (0.000 to 0.004)	
mRS score >2	Conventional model	0.789 (0.765 to 0.812)	0.5925	Ref.	0.0004	Ref.	0.2252
	Conventional model + GA	0.790 (0.766 to 0.813)		0.1867 (0.0840 to 0.2895)		0.0009 (−0.0005 to 0.0023)	
Combined vascular events	Conventional model	0.598 (0.557 to 0.638)	0.6839	Ref.	0.2520	Ref.	0.2060
	Conventional model + GA	0.600 (0.558 to 0.641)		0.0430 (−0.0300 to 0.1230)		0.000 (0.000 to 0.0040)	
1 year							
Stroke recurrence	Conventional model	0.596 (0.562 to 0.629)	0.4698	Ref.	0.2060	Ref.	0.1660
	Conventional model + GA	0.599 (0.566 to 0.633)		0.043 (−0.0420 to 0.1050)		0.0010 (0.000 to 0.0040)	
mRS score >2	Conventional model	0.788 (0.763 to 0.813)	0.0641	Ref.	0.0304	Ref.	0.5293
	Conventional model + GA	0.792 (0.767 to 0.817)		0.123 (0.0116 to 0.2345)		0.0007 (−0.0015 to 0.0030)	
Combined vascular events	Conventional model	0.606 (0.573 to 0.638)	0.4628		0.1200	Ref.	0.1400
	Conventional model + GA	0.609 (0.576 to 0.642)		0.041 (−0.0220 to 0.1060)		0.0010 (0.0000 to 0.0040)	

Abbreviations: CI, confidence interval; GA, glycated albumin; IDI, integrated discrimination improvement; NRI, net reclassification improvement.

^a^ Conventional model: adjusted for age, sex, smoking and drinking status, medical histories (including stroke, hypertension, cardiovascular disease, coronary heart disease, atrial fibrillation, dyslipidemia, diabetes mellitus), the National Institutes of Health Stroke Scale (NIHSS) score, the modified Rankin Scale (mRS) score before stroke, the TOAST (Trial of Org 10172 in Acute Stroke Treatment) classification (including five subtypes of ischemic stroke: large‐artery atherosclerosis, cardioembolism, small‐vessel occlusion, other determined etiologies, and undetermined causes), intravenous thrombolysis therapy, and laboratory tests (including hypersensitive C‐reactive protein, and low‐density lipoprotein).

### 3.4 HbA1c and clinical outcomes

In our study, HbA1c showed the same significant association with GA. Higher HbA1c levels were associated with higher risk of stroke recurrence (adjusted OR, 1.68; 95% CI, 1.26–2.25), poor functional outcomes (adjusted OR, 1.43; 95% CI, 1.01–2.03), and combined vascular events (adjusted OR, 1.62; 95% CI, 1.22–2.15) at both 3 months and 1 year (Table 4). As for the discrimination test, when adding HbA1c to conventional model, there was slight improvement in predicting stroke recurrence (NRI: 4.8%, *p* = 0.0330) and poor functional outcomes (NRI: 15.08%, *p* = 0.0006) at 1 year. There was no statistical significance in discrimination test at 3 months (Table 5). The accuracy, specificity, sensitivity, and predictive values of C‐statistic are shown in Table S3.

**TABLE 4:** HbA1c level of three outcomes at 3 months and 1 year for ischemic stroke patients.

Outcomes	Q1	Q2	Q3	Q4	*p* for trend	Per 1 SD increase	HbA1c as continuous variable
	<5.50%	5.50%–5.91%	5.91%–6.91%	>6.91%			
3 months							
Stroke recurrence							
Case, *n* (%)	48 (4.97)	51 (5.32)	61 (6.29)	67 (6.93)			
Unadjusted	1	1.20 (0.92–1.56)	1.51* (1.16–1.95)	1.71* (1.33–2.20)	<0.0001	1.66* (1.29–2.12)	1.09* (1.05–1.14)
Model 1	1	1.15 (0.88–1.49)	1.41* (1.08–1.83)	1.64* (1.27–2.11)	<0.0001	1.64* (1.28–2.11)	1.09* (1.04–1.14)
Model 2	1	1.02 (0.73–1.41)	1.35 (0.97–1.87)	1.68* (1.11–2.55)	0.0090	1.61* (1.05–2.46)	1.09* (1.01–1.17)
Model 3	1	1.03 (0.73–1.45)	1.31 (0.93–1.86)	1.57* (1.01–2.45)	0.0306	1.44 (0.91–2.29)	1.07 (0.98–1.16)
mRS score >2							
Case, *n* (%)	83 (8.77)	92 (9.80)	128 (13.39)	119 (12.57)			
Unadjusted	1	1.06 (0.88–1.27)	1.18 (0.98–1.43)	1.29* (1.08–1.55)	0.0026	1.13* (1.06–1.19)	1.07* (1.03–1.11)
Model 1	1	0.94 (0.78–1.14)	1.00 (0.83–1.21)	1.17 (0.97–1.41)	0.0587	1.12* (1.05–1.19)	1.07* (1.03–1.11)
Model 2	1	0.83 (0.64–1.09)	0.94 (0.71–1.24)	0.84 (0.59–1.19)	0.4517	1.02 (0.91–1.14)	1.01 (0.95–1.08)
Model 3	1	0.81 (0.61–1.07)	0.96 (0.72–1.28)	0.88 (0.61–1.26)	0.6651	1.04 (0.92–1.17)	1.02 (0.95–1.10)
Combined vascular events^a^							
Case, *n* (%)	49 (5.08)	53 (5.53)	62 (6.39)	68 (7.03)			
Unadjusted	1	1.16 (0.90–1.5)	1.47* (1.14–1.89)	1.64* (1.28–2.09)	<0.0001	1.61* (1.26–2.06)	1.09* (1.04–1.13)
Model 1	1	1.10 (0.85–1.43)	1.36* (1.05–1.75)	1.57* (1.22–2.00)	<0.0001	1.60* (1.25–2.06)	1.09* (1.04–1.13)
Model 2	1	0.98 (0.71–1.34)	1.30 (0.95–1.79)	1.66* (1.10–2.49)	0.0102	1.61* (1.06–2.47)	1.09* (1.01–1.17)
Model 3	1	0.98 (0.70–1.36)	1.26 (0.90–1.76)	1.51 (0.98–2.33)	0.0444	1.39 (0.88–2.21)	1.06 (0.98–1.15)
1 year							
Stroke recurrence							
Case, *n* (%)	66 (6.84)	76 (7.92)	90 (9.28)	106 (10.96)			
Unadjusted	1	1.19 (0.97–1.46)	1.35* (1.10–1.66)	1.61* (1.32–1.96)	<0.0001	1.48* (1.20–1.81)	1.07* (1.03–1.11)
Model 1	1	1.14 (0.93–1.40)	1.27* (1.03–1.56)	1.56* (1.28–1.90)	<0.0001	1.47* (1.20–1.81)	1.07* (1.03–1.11)
Model 2	1	1.11 (0.86–1.43)	1.31* (1.02–1.70)	1.68* (1.26–2.25)	0.0003	1.42* (1.04–1.94)	1.06* (1.01–1.12)
Model 3	1	1.13 (0.86–1.48)	1.36* (1.04–1.79)	1.65* (1.21–2.25)	0.0009	1.34 (0.95–1.88)	1.05 (0.99–1.12)
mRS score >2							
Case, *n* (%)	59 (6.41)	84 (9.15)	94 (10.18)	110 (12.01)			
Unadjusted	1	1.26* (1.02–1.55)	1.19 (0.96–1.48)	1.39* (1.13–1.72)	0.0054	1.14* (1.07–1.22)	1.08* (1.04–1.12)
Model 1	1	1.11 (0.89–1.37)	0.99 (0.79–1.23)	1.27* (1.02–1.57)	0.0742	1.15* (1.07–1.23)	1.08* (1.04–1.13)
Model 2	1	1.12 (0.89–1.60)	1.11 (0.82–1.51)	1.43* (1.01–2.03)	0.0888	1.20* (1.08–1.34)	1.11* (1.04–1.19)
Model 3	1	1.18 (0.87–1.61)	1.09 (0.79–1.51)	1.46* (1.01–2.11)	0.0958	1.21* (1.08–1.36)	1.12* (1.05–1.2)
Combined vascular events^a^							
Case, *n* (%)	69 (7.15)	79 (8.24)	94 (9.69)	112 (11.58)			
Unadjusted	1	1.17 (0.96–1.42)	1.30* (1.07–1.59)	1.55* (1.28–1.88)	<0.0001	1.43* (1.18–1.75)	1.07* (1.03–1.10)
Model 1	1	1.11 (0.91–1.35)	1.21 (0.99–1.48)	1.50* (1.24–1.81)	<0.0001	1.43* (1.17–1.76)	1.07* (1.03–1.10)
Model 2	1	1.11 (0.87–1.42)	1.26 (0.98–1.62)	1.62* (1.22–2.15)	0.0008	1.37* (1.00–1.86)	1.06* (1.00–1.11)
Model 3	1	1.13 (0.87–1.47)	1.30* (1.00–1.69)	1.57* (1.16–2.13)	0.0029	1.27 (0.91–1.77)	1.04 (0.98–1.11)

*Note*: Hazard ratios (HRs) with 95% confidence intervals (CIs) were used for stroke recurrence and combined vascular events; odds ratios (ORs) with 95% CIs were used for modified Rankin Scale (mRS) score >2. Unadjusted model was crude model without adjustment. Model 1 was adjusted for age and sex. Model 2 was adjusted for age, sex, smoking and drinking status, medical histories (including stroke, hypertension, cardiovascular disease, coronary heart disease, atrial fibrillation, dyslipidemia, diabetes mellitus), the National Institutes of Health Stroke Scale (NIHSS) score, the mRS score before stroke, the TOAST (Trial of Org 10172 in Acute Stroke Treatment) classification (including five subtypes of ischemic stroke: large‐artery atherosclerosis, cardioembolism, small‐vessel occlusion, other determined etiologies, and undetermined causes), intravenous thrombolysis therapy, and laboratory tests (including hypersensitive C‐reactive protein, and low‐density lipoprotein). Model 3 was adjusted for age, sex, smoking and drinking status, medical histories (including stroke, hypertension, cardiovascular disease, coronary heart disease, atrial fibrillation, dyslipidemia, diabetes mellitus), the NIHSS score, the mRS score before stroke, the TOAST classification (including five subtypes of ischemic stroke: large‐artery atherosclerosis, cardioembolism, small‐vessel occlusion, other determined etiologies, and undetermined causes), intravenous thrombolysis therapy, and laboratory tests (including hypersensitive C‐reactive protein, and low‐density lipoprotein), the time between the onset of the symptoms and the enrollment, prestroke physical activity, and prestroke medication history (including antiplatelet therapy, anticoagulation therapy, and hypoglycemic treatment).

Abbreviations: GA, glycated albumin; SD, standard deviation.

^a^ Combined vascular events included cardiovascular death, nonfatal myocardial infarction, and nonfatal stroke.

* *p* < 0.05.

**TABLE 5:** Reclassification and disclination statistics for stroke adverse outcomes when adding to HbA1c levels.

Clinical outcomes	Model	C‐statistic		NRI		IDI	
		Estimate (95% CI)	*p* value	Estimate (95% CI)	*p* value	Estimate (95% CI)	*p* value
3 months							
Stroke recurrence	Conventional model^a^	0.647 (0.617 to 0.676)	0.1242	Ref.	0.0600	Ref.	0.0660
	Conventional model + HbA1c	0.653 (0.624 to 0.682)		0.0640 (−0.0010 to 0.1160)		0.0010 (0.0000 to 0.0030)	
mRS score >2	Conventional model	0.817 (0.800 to 0.834)	0.1318	Ref.	0.3422	Ref.	0.7690
	Conventional model + HbA1c	0.818 (0.801 to 0.834)		0.0369 (−0.0392 to 0.1129)		−0.0001 (−0.0008 to 0.0006)	
Combined vascular events	Conventional model	0.654 (0.625 to 0.682)	0.1188	Ref.	0.0530	Ref.	0.0930
	Conventional model + HbA1c	0.659 (0.631 to 0.688)		0.0600 (−0.0070 to 0.1140)		0.0010 (0.0000 to 0.0030)	
1 year							
Stroke recurrence	Conventional model	0.630 (0.605 to 0.653)	0.0352	Ref.	0.0330	Ref.	0.1060
	Conventional model + HbA1c	0.635 (0.612 to 0.659)		0.0480 (0.0030 to 0.0950)		0.0010 (0.0000 to 0.0030)	
mRS score >2	Conventional model	0.795 (0.776 to 0.815)	0.0013	Ref.	0.0006	Ref.	0.5028
	Conventional model + HbA1c	0.802 (0.783 to 0.821)		0.1508 (0.0651 to 0.2366)		0.0008 (−0.0015 to 0.0030)	
Combined vascular events	Conventional model	0.637 (0.614 to 0.661)	0.0468	Ref.	0.0860	Ref.	0.2590
	Conventional model + HbA1c	0.642 (0.619 to 0.665)		0.0430 (−0.0130 to 0.0920)		0.0000 (0.0000 to 0.0020)	

Abbreviations: CI, confidence interval; HbA1c, glycosylated hemoglobin; IDI, integrated discrimination improvement; NRI, net reclassification improvement.

^a^ Conventional model: adjusted for age, sex, smoking and drinking status, medical histories (including stroke, hypertension, cardiovascular disease, coronary heart disease, atrial fibrillation, dyslipidemia, diabetes mellitus), the National Institutes of Health Stroke Scale (NIHSS) score, the modified Rankin Scale (mRS) score before stroke, the TOAST (Trial of Org 10172 in Acute Stroke Treatment) classification (including five subtypes of ischemic stroke: large‐artery atherosclerosis, cardioembolism, small‐vessel occlusion, other determined etiologies, and undetermined causes), intravenous thrombolysis therapy, and laboratory tests (including hypersensitive C‐reactive protein, and low‐density lipoprotein).

## 4 DISCUSSION

The large prospective multicenter cohort study including 3861 participants found a positive relationship between GA level and adverse stroke outcomes. The baseline serum level of GA after stroke onset was positively associated with recurrence, poor functional outcomes, and combined cardiovascular events at 3 months and 1 year. The effect of GA on stroke outcome remained after adjusting for other risk factors. In subgroup analyses, higher GA levels were associated with poor short‐term functional outcomes only in patients without diabetes. Higher HbA1c levels were associated with higher risk of stroke recurrence, poor functional outcomes, and combined vascular events at both 3 months and 1 year. Adding GA level into the conventional model resulted in an 18.67% increase at 3 months and a 12.3% increase at 1 year in predicting poor functional outcomes. Adding HbA1c to conventional model, there was a slight improvement in predicting stroke recurrence (NRI: 4.8%) and poor functional outcomes (NRI: 15.08%) at 1 year.

Our study found that GA was associated with stroke recurrence and poor functional outcomes, which seriously affected the quality of life. Glycemic status is associated with stroke occurrence, progression, and outcomes. GA could reflect glycemic control more accurately 2–4 weeks before stroke onset. Previous studies have also shown that higher glycosylated albumin levels were associated with stroke incidence and adverse stroke outcomes. For the healthy population, a Japanese community cohort study showed that higher GA levels were positively associated with the incidence of stroke.^10^ Regarding early neurological deterioration, a single‐center retrospective study indicated that more deterioration events occurred in the higher GA group within the first 7 days after stroke onset.^11^ In another cross‐sectional study conducted by the same team, a higher GA level was associated with a higher NIHSS score and larger infarct volume on admission.^12^ However, the smaller sample size, single‐center design, and lack of short‐term or long‐term follow‐up limited the applicability and generalizability of these studies. A subgroup analysis of the Clopidogrel in High‐Risk Patients with Acute Nondisabling Cerebrovascular Events (CHANCE) trial demonstrated the predictive role of GA on the outcome of dual or single antiplatelet therapy,^13^ highlighting the importance of glycemic stabilization for the effectiveness of antiplatelet therapy. In a total of 1168 Acute Ischemic Stroke patients, after adjusting for multiple covariates, patients in the higher GA group (GA *≥*16%) had a 1.4‐fold risk of having unfavorable mRS scores (95% CI, 1.02–1.847) at the 3‐month follow‐up.^14^ Our study was a prospective multicenter study with a large sample size and long‐term follow‐up. We found a positive association between GA and adverse stroke outcomes at both the 3‐month and 1‐year follow‐ups; moreover, the association persisted after adjusting for other confounding factors. Furthermore, we conducted a discrimination test and found that GA could improve the ability of conventional model to predict the risk of poor functional outcomes 3 months and 1 year after stroke onset, which has rarely been confirmed before.

Clinical predictive models are a quantitative risk and benefit assessment tool that can provide more objective and accurate information for doctors. The evaluation and verification of the effectiveness of prediction models are the key to statistical analysis. C‐statistic, NRI, and IDI are all analytical metrics to evaluate the effectiveness of prediction models. C‐statistic is equivalent to the area under the Receiver Operator Characteristic curve (AUC) in regression analysis that can assess the performance of prediction models. However, when it comes to evaluating the improvement of predictive performance after incorporating a new variable, the improvement of C‐statistic/AUC is always small. NRI and IDI evaluate the model based on the increase in the proportion and probability of predicting correct outcome, which can amplify the subtle differences between the two models.^15^

In our study, we found that adding GA to conventional model could improve the ability of the conventional model to predict the risk of poor functional outcomes at 3 months and 1 year after stroke onset. Higher levels of GA after stroke onset suggested poor glycemic management before stroke. For one thing, long‐term hyperglycemia can lead to vascular endothelial function impairment, promoting the formation and progression of atherosclerosis.^14^ Furthermore, for patients with normal blood sugar level, short‐term blood sugar increase before stroke would produce higher levels of oxidative stress and endothelial dysfunction than persistent hyperglycemia.^16^ Atherosclerosis and endothelial dysfunction are both significant factors leading to poor functional outcomes of stroke.

HbA1c reflects the glycemia level over the past 2–3 months, and its predictive effect on adverse stroke outcomes has been widely explained. A retrospective analysis of 7380 patients showed that high HbA1c levels increased the risk of short‐ and long‐term poor functional outcomes after ischemic stroke onset (3‐month OR, 1.299, 95% CI, 1.098–1.535; 1‐year OR, 1.181, 95% CI, 0.952–1.465).^17^ Our study revealed the same findings as previous studies. Higher HbA1c levels were associated with higher risk of stroke recurrence (adjusted OR, 1.68; 95% CI, 1.26–2.25), poor functional outcomes (adjusted OR, 1.43; 95% CI, 1.01–2.03), and combined vascular events (adjusted OR, 1.62; 95% CI, 1.22–2.15) at both 3 months and 1 year. As for the discrimination test, when adding HbA1c to conventional model, there was slight improvement in predicting stroke recurrence (NRI: 4.8%, *p* = 0.0330) and poor functional outcomes (NRI: 15.08%, *p* = 0.0006) at 1 year. Both GA and HbA1c are blood indicators of glycemic control. As for availability, HbA1c is widely used in clinical diagnosis and treatment, and the American Diabetes Association (ADA), Federal Drug Association (FDA), and the Canadian Diabetes Association (CDA) have accepted HbA1c as an approved indicator for long‐term glycemic control.^18^ The commonly used detection methods for HbA1c are latex agglutination reaction and high‐performance liquid chromatography (HPLC). The latter (referring to HPLC) has been standardized according to The Japan Diabetes Society.^19^ However, GA is not commonly available at clinical sites and community monitoring because of cost of the equipment and complexity of procedure.^20^ As for the practicality, the ability to predict the prognosis of ischemic stroke depends on which indicator can better reflect glycemic control. HbA1c reflects the glycemia level over the past 2–3 months; however, it does not reflect glycemic control accurately under conditions with rapid changes in the lifespan of red blood cells. In addition, it is known that in hematologic disorders (such as anemia and variant hemoglobin), abnormal HbA1c levels are observed.^21^ GA, which is not influenced by changes in the lifespan of erythrocytes, is thought to be an alternative indicator for glycemic control.^22^ Moreover, GA itself is correlated with stroke prognosis, as demonstrated in previous studies and ours, which cannot be ignored. Therefore, it is important for clinician to be aware of different indicators for different situations.

Previous studies also found that the serum GA level was positively associated with cardiovascular disease. Wang et al. reported that the GA/HbA1c ratio was significantly associated with the risk of cardiovascular mortality in a nationally representative cohort of American adults.^23^ Another small cohort study conducted in Japan showed that GA could predict the occurrence of coronary artery disease (CAD) and could be superior to HbA1c.^24^ These studies were conducted in cardiovascular patients. Our study focused on cardiovascular events in stroke patients, which have received less attention. We found that in stroke patients, GA was positively associated with the incidence of combined vascular events. The potential mechanism may be related to the atherosclerotic susceptibility of elevated GA. GA itself can also promote atherosclerosis.^25^ The occurrence and development of atherosclerosis are related to oxidative stress, which can cause vascular endothelial damage and promote the formation of lipid plaques.^26^ The glycosylation of albumin causes the impairment of the antioxidant activity of albumin, thereby accelerating the process of atherosclerosis.^27^

Interestingly, after classification by diabetes history, adverse effects of GA on stroke outcomes were observed only in nondiabetic patients (adjusted OR, 1.82, 95% CI, 1.12–2.94). For poor functional outcomes, the adjusted OR was 1.62 (95% CI, 1.05–2.50) at 3 months and 1.82 (95% CI, 1.12–2.94) at 1 year. The same results were found in a Korean cohort study. They reported that GA was independently associated with unfavorable short‐term outcomes only in nondiabetic patients and not in diabetic patients.^14^ Previous studies have shown that elevated blood sugar on admission leads to a higher risk of death at 30 days after stroke in nondiabetic patients.^28^ The possible mechanism of the association between GA and stroke outcome may be related to oxidative stress caused by glycemic variability. GA is a blood marker for monitoring short‐term glycemic fluctuation.^24^ Preclinical and clinical studies have shown that exposure to rapid glycemic fluctuation produced higher levels of oxidative stress and endothelial dysfunction than persistent hyperglycemia, thereby worsening microangiopathy and macroangiopathy.^16^, ^29^, ^30^, ^31^, ^32^ Excessive reactive oxygen species (ROS) and nicotinamide adenine dinucleotide phosphate (NADPH) produced by hyperglycemia can cause neuronal death,^33^ ultimately resulting in increased infarct size and poor outcomes.^12^, ^34^

The major strength of our project is that this is a multicenter, prospective cohort study that has by far the largest number of cases and has a long‐term follow‐up. In addition, we adjusted for as many available confounding factors as possible and conducted subgroup analyses of age, sex, and history of diabetes for sensitivity analyses. However, there are also several limitations that should be noted. First, there were fewer female patients in CNSR‐III, which may have an impact on stroke outcomes. The female proportion is also underrepresented in other Chinese study cohorts because Asian female patients may be less likely to seek medical assistance.^35^ Second, we did not adjust for other factors that affect the state of protein metabolism, such as liver cirrhosis, thyroid dysfunction, and nephrotic syndrome. We did not have enough cases to cover these disease states. Finally, we did not measure blood glucose changes during hospitalization, which reflects the rate of glycemic variability during hospitalization. And considering the exact time of the outcomes event is hard to predict accurately and the monitoring equipment limitations, we were not able to detect GA before the event occurred or monitor the level of GA continuously. Hence, the next step is to analyze GA levels and stroke outcomes in a larger population, including different disease states that affect protein metabolism and continuous blood glucose and GA measurements.

## 5 CONCLUSIONS

In conclusion, our study demonstrated that elevated GA levels in serum were associated with stroke adverse outcomes, including stroke recurrence, poor functional outcomes, and combined vascular events in patients with ischemic stroke or TIA. Subgroup analysis showed a stronger association in nondiabetic patients. This suggests that for nondiabetic patients with high GA levels, the occurrence of stroke adverse outcomes should be monitored and more effective medical interventions should be performed.

## AUTHOR CONTRIBUTIONS

**Jiawen Mao:** Investigation; writing—original draft. **Meng Wang:** Methodology; formal analysis; investigation; writing–review and editing; funding acquisition. **Chunjuan Wang:** Investigation; writing–review and editing. **Hongqiu Gu:** Formal analysis; investigation; writing–review and editing. **Xia Meng:** Writing—review and editing. **Yong Jiang:** Writing—review and editing. **Xin Yang:** Writing–review and editing. **Jing Zhang:** Writing–review and editing. **Yunyun Xiong:** Writing–review and editing. **Xingquan Zhao:** Writing–review and editing. **Liping Liu:** Writing—review and editing. **Yilong Wang:** Writing—review and editing. **Yongjun Wang:** Writing—review and editing. **Zixiao Li:** Investigation; writing—review and editing; resources; supervision; project administration; funding acquisition. **Bihong Zhu:** Investigation; writing–review and editing; resources; supervision; project administration; funding acquisition.

## FUNDING INFORMATION

This study was supported by grants from the General Project of Science and Technology Plan of Beijing Municipal Education Commission (KM202310025012), Natural Science Foundation of Beijing Municipality (Z200016), National Natural Science Foundation of China (82171270, 82301481, 92046016), National Key Research and Development Program of China (2022YFC2504900, 2022YFC25049002), CAMS Innovation Fund for Medical Sciences (2019‐I2M‐5‐029), Beijing Hospitals Authority (PX2021024), Nature Science Foundation of Capital Medical University (PYZ22111), and Cerebrovascular Disease Youth Innovation Fund (Z‐2016‐20‐2201).

## CONFLICT OF INTEREST STATEMENT

The authors declare no conflicts of interest.

## Supporting information


**Data S1:** Supporting Information.

## Data Availability

Data from study are available from the China National Clinical Research Center for Neurological Diseases. Maintenance of data availability is guaranteed by the corresponding author (contact: lizixiao2008@hotmail.com or bihong430@163.com).
