# Supplementary material for: Glycated albumin levels are associated with adverse stroke outcomes in patients with acute ischemic stroke in China
Source: J Diabetes. 2024 Sep 12;16(9):e13600. doi: 10.1111/1753-0407.13600 (PMC11391381; doi:10.1111/1753-0407.13600)
Supplement: Supplementary file 1 — Data S1: Supporting Information. [file JDB-16-e13600-s001.docx]

**Supplement Table 1. Differences of three outcomes at 3 months and 1 year in subgroup analysis.**

| **Outcomes** | **Q1** | **Q2** | **Q3** | **Q4** | ***p* for interaction** |
| --- | --- | --- | --- | --- | --- |
| 3 months |  |  |  |  |  |
| Stroke recurrence |  |  |  |  |  |
| History of diabetes-No | 1 | 1.01  (0.66, 1.54) | 1.03  (0.66, 1.62) | 1.60  (0.99, 2.60) | 0.2545 |
| History of diabetes-Yes | 1 | 0.61  (0.08, 4.38) | 2.08  (0.47, 9.23) | 1.62  (0.38, 6.81) | 0.2545 |
| Age ≤ 65 years | 1 | 1.34  (0.77, 2.33) | 1.95  (1.13, 3.38) | 2.13  (1.17, 3.88) | 0.0626 |
| Age > 65 years | 1 | 0.62  (0.34, 1.16) | 0.64  (0.36, 1.15) | 0.89  (0.47, 1.69) | 0.0626 |
| Sex-Male | 1 | 0.79  (0.39, 1.62) | 1.04  (0.53, 2.06) | 1.08  (0.51, 2.30) | 0.9120 |
| Sex-Female | 1 | 1.08  (0.65, 1.79) | 1.25  (0.75, 2.09) | 1.58  (0.92, 2.73) | 0.9120 |
| mRS>2 |  |  |  |  |  |
| History of diabetes-No | 1 | 1.10  (0.76, 1.59) | 1.54  (1.07, 2.22) | 1.62  (1.05, 2.50) | 0.8172 |
| History of diabetes-Yes | 1 | 0.46  (0.12, 1.78) | 0.86  (0.29, 2.59) | 0.79  (0.28, 2.23) | 0.8172 |
| Age ≤ 65 years | 1 | 1.52  (0.94, 2.44) | 1.90  (1.17, 3.10) | 1.61  (0.93, 2.79) | 0.1552 |
| Age > 65 years | 1 | 0.73  (0.43, 1.23) | 1.20  (0.74, 1.93) | 1.26  (0.75, 2.14) | 0.1552 |
| Sex-Male | 1 | 0.89  (0.47, 1.69) | 1.49  (0.83, 2.68) | 1.49  (0.79, 2.84) | 0.5515 |
| Sex-Female | 1 | 1.15  (0.76, 1.76) | 1.53  (1.00, 2.34) | 1.50  (0.94, 2.41) | 0.5515 |
| Combined vascular events |  |  |  |  |  |
| History of diabetes-No | 1 | 1.02  (0.67, 1.54) | 1.02  (0.65, 1.58) | 1.56  (0.96, 2.52) | 0.2725 |
| History of diabetes-Yes | 1 | 0.60  (0.08, 4.35) | 2.07  (0.47, 9.19) | 1.66  (0.40, 6.99) | 0.2725 |
| Age ≤ 65 years | 1 | 1.34  (0.77, 2.33) | 1.95  (1.13, 3.38) | 2.13  (1.17, 3.88) | 0.0591 |
| Age > 65 years | 1 | 0.67  (0.37, 1.21) | 0.64  (0.36, 1.14) | 0.90  (0.48, 1.68) | 0.0591 |
| Sex-Male | 1 | 0.75  (0.37, 1.52) | 0.97  (0.50, 1.90) | 1.02  (0.48, 2.14) | 0.8342 |
| Sex-Female | 1 | 1.13  (0.69, 1.86) | 1.26  (0.76, 2.10) | 1.59  (0.93, 2.73) | 0.8342 |
| 1 year |  |  |  |  |  |
| Stroke recurrence |  |  |  |  |  |
| History of diabetes-No | 1 | 1.15  (0.80, 1.64) | 1.19  (0.82, 1.73) | 1.69  (1.12, 2.54) | 0.3753 |
| History of diabetes-Yes | 1 | 0.46  (0.10, 2.08) | 1.38  (0.47, 4.07) | 1.37  (0.49, 3.81) | 0.3753 |
| Age ≤ 65 years | 1 | 1.25  (0.79, 1.97) | 1.79  (1.14, 2.82) | 2.03  (1.25, 3.32) | 0.2063 |
| Age > 65 years | 1 | 0.90  (0.54, 1.51) | 0.86  (0.52, 1.42) | 1.22  (0.71, 2.10) | 0.2063 |
| Sex-Male | 1 | 0.86  (0.47, 1.58) | 1.05  (0.59, 1.88) | 1.51  (0.82, 2.80) | 0.8754 |
| Sex-Female | 1 | 1.21  (0.80, 1.83) | 1.36  (0.89, 2.09) | 1.52  (0.96, 2.40) | 0.8754 |
| mRS>2 |  |  |  |  |  |
| History of diabetes-No | 1 | 1.51  (1.00, 2.26) | 1.41  (0.92, 2.16) | 1.82  (1.12, 2.94) | 0.1024 |
| History of diabetes-Yes | 1 | 0.22  (0.05, 0.90) | 0.45  (0.16, 1.32) | 0.58  (0.22, 1.51) | 0.1024 |
| Age ≤ 65 years | 1 | 1.72  (0.96, 3.07) | 1.62  (0.87, 3.02) | 2.28  (1.20, 4.32) | 0.7181 |
| Age > 65 years | 1 | 1.12  (0.67, 1.88) | 1.18  (0.71, 1.95) | 1.43  (0.83, 2.47) | 0.7181 |
| Sex-Male | 1 | 0.89  (0.47, 1.68) | 0.98  (0.53, 1.81) | 1.52  (0.79, 2.89) | 0.5115 |
| Sex-Female | 1 | 1.54  (0.94, 2.50) | 1.48  (0.90, 2.44) | 1.69  (0.98, 2.90) | 0.5115 |
| Combined vascular events |  |  |  |  |  |
| History of diabetes-No | 1 | 1.13  (0.80, 1.60) | 1.19  (0.83, 1.72) | 1.60  (1.07, 2.40) | 0.4661 |
| History of diabetes-Yes | 1 | 0.46  (0.10, 2.07) | 1.39  (0.47, 4.09) | 1.51  (0.55, 4.19) | 0.4661 |
| Age ≤ 65 years | 1 | 1.21  (0.78, 1.90) | 1.70  (1.09, 2.66) | 1.96  (1.21, 3.17) | 0.3158 |
| Age > 65 years | 1 | 0.92  (0.55, 1.52) | 0.91  (0.56, 1.47) | 1.28  (0.75, 2.16) | 0.3158 |
| Sex-Male | 1 | 0.81  (0.45, 1.48) | 1.03  (0.59, 1.82) | 1.47  (0.81, 2.69) | 0.8285 |
| Sex-Female | 1 | 1.21  (0.81, 1.82) | 1.33  (0.88, 2.01) | 1.51  (0.97, 2.36) | 0.8285 |

Adjusted for age, sex, smoking and drinking status, medical histories (including stroke, hypertension, cardiovascular disease, coronary heart disease, atrial fibrillation, dyslipidemia, diabetes mellitus), the NIHSS score, the mRS score before stroke, the TOAST classification (including five subtypes of ischemic stroke: large-artery atherosclerosis, cardioembolism, small-vessel occlusion, other determined etiologies, and undetermined causes), intravenous thrombolysis therapy and laboratory tests (including hypersensitive C-reactive protein and low density lipoprotein).

GA, glycated albumin; mRS, the modified Rankin Scale; CI, confidence interva

| **Clinical outcomes** | **Model** | **C-statistic** | | | | | | | | | |
| --- | --- | --- | --- | --- | --- | --- | --- | --- | --- | --- | --- |
|  |  | **Accuracy**  **(%)** | | **Specificity (%)** | | **Sensitivity (%)** | | **Positive predictive values (%)** | | **Negative predictive values (%)** | |
| 3 months |  |  | |  | |  | |  | |  | |
| Stroke recurrence | Conventional  Model^a^ | 66.78 | | 67.97 | | 46.95 | | 80.78 | | 95.53 | |
|  | Conventional model+GA | 64.87 | | 65.63 | | 52.11 | | 83.33 | | 95.81 | |
| mRS>2 | Conventional  model | 75.73 | | 76.78 | | 67.16 | | 26.09 | | 95.04 | |
|  | Conventional model+GA | 78.19 | | 79.87 | | 64.43 | | 28.09 | | 94.84 | |
| Combined vascular events | Conventional  model | 70.82 | | 72.49 | | 43.58 | | 8.87 | | 95.44 | |
|  | Conventional model+GA | 64.47 | | 65.28 | | 51.38 | | 8.33 | | 95.62 | |
| 1 year |  |  |  | |  | |  | |  | |  |
| Stroke recurrence | Conventional  model | 63.41 | | 64.62 | | 50.47 | | 11.73 | | 93.33 | |
|  | Conventional model+GA | 65.19 | | 66.68 | | 49.22 | | 12.10 | | 93.37 | |
| mRS>2 | Conventional  model | 70.01 | | 69.61 | | 73.94 | | 19.77 | | 96.35 | |
|  | Conventional model+GA | 69.34 | | 68.69 | | 75.76 | | 19.69 | | 96.55 | |
| Combined vascular events | Conventional  model | 51.14 | | 49.55 | | 67.36 | | 11.60 | | 93.92 | |
|  | Conventional model+GA | 74.91 | | 78.42 | | 39.17 | | 15.14 | | 92.92 | |

**Supplement table 2. The performance metrics for C-statistic of GA model.**

GA, glycated albumin; mRS, the modified Rankin Scale;

^a^ Conventional model: adjusted for age, sex, smoking and drinking status, medical histories (including stroke, hypertension, cardiovascular disease, coronary heart disease, atrial fibrillation, dyslipidemia, diabetes mellitus), the NIHSS score, the mRS score before stroke, the TOAST classification (including five subtypes of ischemic stroke: large-artery atherosclerosis, cardioembolism, small-vessel occlusion, other determined etiologies, and undetermined causes), intravenous thrombolysis therapy and laboratory tests (including hypersensitive C-reactive protein and low density lipoprotein).

| **Clinical outcomes** | **Model** | **C-statistic** | | | | | | | | | |
| --- | --- | --- | --- | --- | --- | --- | --- | --- | --- | --- | --- |
|  |  | **Accuracy**  **(%)** | | **Specificity (%)** | | **Sensitivity (%)** | | **Positive predictive values (%)** | | **Negative predictive values (%)** | |
| 3 months |  |  | |  | |  | |  | |  | |
| Stroke recurrence | Conventional  Model^a^ | 79.54 | | 61.24 | | 60.06 | | 11.43 | | 95.72 | |
|  | Conventional model+HbA1c | 76.92 | | 59.82 | | 64.74 | | 10.67 | | 95.77 | |
| mRS>2 | Conventional  model | 70.26 | | 73.80 | | 75.52 | | 21.82 | | 95.74 | |
|  | Conventional model+HbA1c | 75.89 | | 72.67 | | 76.92 | | 24.80 | | 95.15 | |
| Combined vascular events | Conventional  model | 77.55 | | 67.07 | | 56.50 | | 11.84 | | 95.80 | |
|  | Conventional model+HbA1c | 74.81 | | 57.58 | | 67.37 | | 11.05 | | 95.85 | |
| 1 year |  |  |  | |  | |  | |  | |  |
| Stroke recurrence | Conventional  model | 55.02 | | 62.98 | | 56.95 | | 11.26 | | 94.16 | |
|  | Conventional model+HbA1c | 74.26 | | 49.48 | | 71.36 | | 14.38 | | 93.53 | |
| mRS>2 | Conventional  model | 67.58 | | 70.02 | | 74.64 | | 17.84 | | 96.98 | |
|  | Conventional model+HbA1c | 71.11 | | 72.63 | | 74.10 | | 19.23 | | 96.77 | |
| Combined vascular events | Conventional  model | 57.97 | | 63.44 | | 56.89 | | 12.43 | | 93.96 | |
|  | Conventional model+HbA1c | 73.38 | | 52.26 | | 68.56 | | 15.32 | | 93.36 | |

**Supplement table 3. The performance metrics for C-statistic of HbA1c model.**

HbA1c, glycosylated hemoglobin; mRS, the modified Rankin Scale;

^a^ Conventional model: adjusted for age, sex, smoking and drinking status, medical histories (including stroke, hypertension, cardiovascular disease, coronary heart disease, atrial fibrillation, dyslipidemia, diabetes mellitus), the NIHSS score, the mRS score before stroke, the TOAST classification (including five subtypes of ischemic stroke: large-artery atherosclerosis, cardioembolism, small-vessel occlusion, other determined etiologies, and undetermined causes), intravenous thrombolysis therapy and laboratory tests (including hypersensitive C-reactive protein and low density lipoprotein).

**
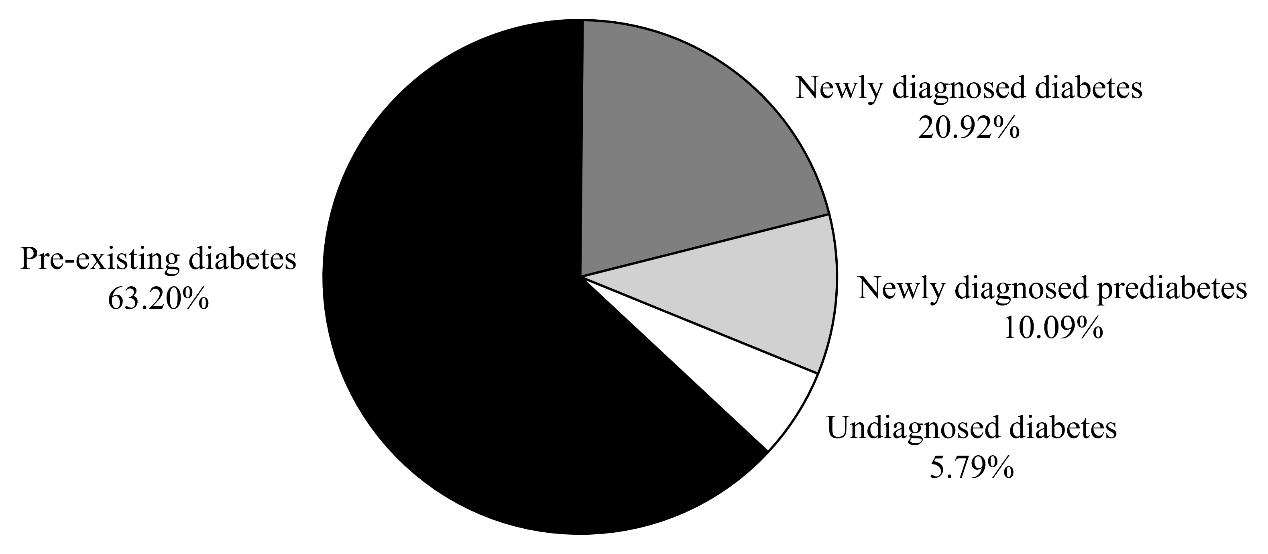
Supplement figure 1. The proportion of diabetic population in Q4 group of GA.** According to the diagnostic criteria for diabetes: HbA1c ≥ 6.5% was diagnosed as diabetes; 5.7% ≤ HbA1c < 6.5% was diagnosed as prediabetes; HbA1c < 5.7% was diagnosed as nondiabetic. HbA1c data were available for 674 patients out of 967 patients in the Q4 group. 426 (63.20%) patients had pre-existing diabetes. There were 141 (20.92%) patients newly diagnosed diabetes and 68 (10.09%) patients newly diagnosed prediabetes, who denied any history of diabetes. There were 39 (5.79%) patients undiagnosed diabetes.
